# Patient-specific COVID-19 resource utilization prediction using fusion AI model

**DOI:** 10.1038/s41746-021-00461-0

**Published:** 2021-06-03

**Authors:** Amara Tariq, Leo Anthony Celi, Janice M. Newsome, Saptarshi Purkayastha, Neal Kumar Bhatia, Hari Trivedi, Judy Wawira Gichoya, Imon Banerjee

**Affiliations:** 1 Department of Biomedical Informatics, School of Medicine, Emory University, Atlanta, GA USA; 2 Massachusetts Institute of Technology, Boston, MA USA; 3 Division of Pulmonary, Critical Care and Sleep Medicine, Beth Israel Deaconess Medical Center, Boston, MA USA; 4 Harvard Medical School, Boston, MA USA; 5 Department of Radiology and Imaging Sciences, School of Medicine, Emory University, Atlanta, GA USA; 6 School of Informatics Computing, Indiana University Purdue University, Indianapolis, IN USA; 7 Department of Medicine, School of Medicine, Emory University, Atlanta, GA USA

**Keywords:** Machine learning, Predictive medicine, Data mining

## Abstract

The strain on healthcare resources brought forth by the recent COVID-19 pandemic has highlighted the need for efficient resource planning and allocation through the prediction of future consumption. Machine learning can predict resource utilization such as the need for hospitalization based on past medical data stored in electronic medical records (EMR). We conducted this study on 3194 patients (46% male with mean age 56.7 (±16.8), 56% African American, 7% Hispanic) flagged as COVID-19 positive cases in 12 centers under Emory Healthcare network from February 2020 to September 2020, to assess whether a COVID-19 positive patient’s need for hospitalization can be predicted at the time of RT-PCR test using the EMR data prior to the test. Five main modalities of EMR, i.e., demographics, medication, past medical procedures, comorbidities, and laboratory results, were used as features for predictive modeling, both individually and fused together using late, middle, and early fusion. Models were evaluated in terms of precision, recall, F1-score (within 95% confidence interval). The early fusion model is the most effective predictor with 84% overall F1-score [CI 82.1–86.1]. The predictive performance of the model drops by 6 % when using recent clinical data while omitting the long-term medical history. Feature importance analysis indicates that history of cardiovascular disease, emergency room visits in the past year prior to testing, and demographic factors are predictive of the disease trajectory. We conclude that fusion modeling using medical history and current treatment data can forecast the need for hospitalization for patients infected with COVID-19 at the time of the RT-PCR test.

## Introduction

Multiple waves of SARS-CoV-2 virus infections threaten to overwhelm the healthcare system^1^. A third of all hospitalized COVID-19 patients require admission and management in an intensive care unit (ICU)^2^ to manage complications like acute respiratory distress syndrome (ARDS), secondary sepsis, and multi-organ failure^3^. Predictors of poor outcome and need for assisted ventilation include clinical and laboratory markers like D-dimer levels and SOFA score, and demographic features such as older age and ethnicity^3^. Currently, there is no quantitative criterion that combines clinical and laboratory-based markers to predict the likely level of care required for a given patient at the time of COVID-19 testing. Such a predictive model would allow resource planning by understanding potential hospitalization requirements, especially as testing is distributed out of hospitals.

**Fig. 1:**
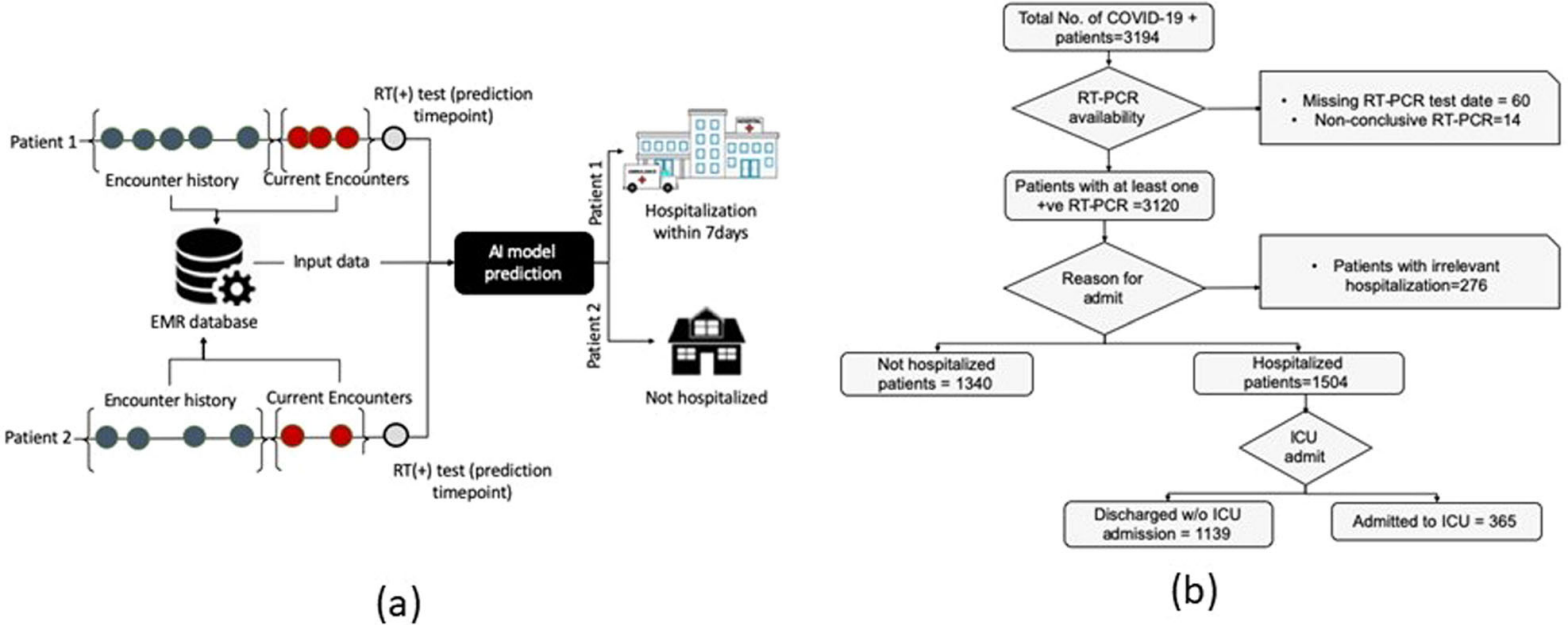
Study design. **a** Proposed AI model decision point shows the prediction of two patients with distinct outcomes. **b** CONSORT diagram for Cohort selection process including decision nodes and a number of excluded cases.

Much of the literature regarding predictive modeling for COVID-19 patients deal with either mortality prediction^4–6^, or analysis of risk factors for mortality^7^. Instead, our work focuses on predicting the probability of future hospitalization at the time of COVID-19 testing (Fig. 1a). This is in contrast to several recent papers that focus on critical event prediction such as ICU admission^8^ and mechanical ventilation^9^ at the time of presentation to the emergency department. Recently published systematic review of COVID19 related prediction models^10^ mentions only three studies related to hospitalization risk prediction (see Supplementary Note 1 for detailed limitations of previously published studies). The major limitation of the existing work, including the studies mentioned by Wynants et. al.^10^, is the use of a narrow feature selection based on expert opinion or published literature^5,6,8,9,11–13^. We overcome this limitation by training multiple machine learning architectures, including multi-branched deep dense network, for the targeted prediction task, using all the data captured in the electronic medical record (EMR) prior to COVID-19 infection. We use interval-based feature representation for medications, comorbidities, past procedures, and laboratory results to ensure that information collected at different time intervals is given due importance by our predictive models. Compared to pre-selected features, we include as many EMR variables as possible, filtering features based on automatic methods while relying on experts to provide intuitive representation or group structure for large features set. We evaluate the predictive performance of each part of the EMR data (demographic information, medication, past procedures, comorbidities, and laboratory results) as well as multiple fusion models that integrate the feature space^14^.

## Results

### Performance of fusion models

Table 1 reports the class-wise and aggregated (weighted average) precision, recall, and F-score^15^ as well as confidence interval (95% confidence) for distinguishing between hospitalization and self-isolation on a held-out set of 569 unique patients. We compare the performance of our fusion models against the performance of individual source classifiers. Results demonstrate that fusing multiple data sources from EMR increases the performance beyond the performance of any individual source. Early fusion is the best performing model with 84 overall F1-score [CI 82.1–86.1] and 85 F1-score for classifying patients who will need hospitalization within 7 days of RT-PCR testing. Late (83 F1-score) and middle fusion (82 F1-score) models also come very close to the performance of the early fusion model.

**Table 2:** Stratified patient characteristics.

Variables	Total cohort (2844 patients)	Train (2275 patients)	Test (569 patients)
AGE, mean(SD)	55.6 (17.9)	55.5 (18.0)	55.7 (17.9)
GENDER [mean age/std]			
Male	1470 (46%) [56.7 (16.8)]	1115 (46%) [56.8 (17.0)]	254 (42%) [56.5 (16.2)]
Female	1719 (54%) [54.5 (18.8)]	1298 (54%) [54.5 (18.8)]	351 (58%) [55.2 (19.1)]
Race			
African American	1678 (56.4%)	1357 (56.1%)	321 (54.4%)
Caucasian/White	593 (19.7%)	474 (19.6%)	119 (19.7%)
Asian	79 (2.6%)	62 (2.6%)	17 (2.8%)
American Indian or Alaska Native	11 (0.4%)	6 (0.3%)	5(0.8%)
Multiple	10 (0.3%)	6 (0.3%)	4 (0.7%)
Native Hawaiian Pacific Islander	6 (0.2%)	2 (0.1%)	4 (0.7%)
Unknown	638 (21.1%)	511 (21.1%)	127 (21.0%)
Ethnic group			
Hispanic or Latino	233 (7.7%)	188 (7.8%)	45 (7.4%)
Non-Hispanic or Latino	2131 (70.5%)	1706 (70.6%)	425 (70.3%)
Unknown	659 (21.8)	524 (21.7%)	135 (22.3%)
Comorbidities			
Respiratory disease	1799 (59.5%)	1435 (59.3%)	364 (60.2%)
Hypertension	1372 (45.4%)	1092 (45.2%)	280 (46.3%)
Renal disease	1016 (33.6%)	806 (33.3%)	210 (34.7%)
Diabetes	467 (15.4%)	380 (15.7%)	87 (14.4%)

**Table 1:** Performance for binary classification models with hospitalization and non-hospitalization as two targets, in terms of class-wise and aggregated (weighted average) precision, recall, and *F*-score; C.I. (95% confidence) was computed using bootstrapping over 1000 iterations with random samples.

		Precision	Recall	F1-score	Number of samples
Demographics	Non hospitalization	68	63	65	277
	Hospitalization	71	74	72	328
	Overall	69	69	69	
	C.I.	66.8–71.6	66.9–71.7	66.8–71.6	
Prescriptions	Non hospitalization	62	86	72	277
	Hospitalization	82	55	66	328
	Overall	73	69	69	
	C.I.	71.2–75.5	67.2–71.9	66.7–71.4	
ICD-9	Non hospitalization	74	84	79	277
	Hospitalization	85	75	80	328
	Overall	80	79	79	
	C.I.	78.1–82.0	77.1–81.4	77.2–81.4	
CPT	Non hospitalization	74	83	78	277
	Hospitalization	84	75	79	328
	Overall	79	79	79	
	C.I.	77.1–81.6	76.5–81.0	76.6–81.1	
Laboratory test results	Non hospitalization	72	78	75	277
	Hospitalization	80	75	77	328
	Overall	76	76	76	
	C.I.	74.1–78.8	74.0–78.8	74.4–79.1	
Late fusion	Non hospitalization	84	77	81	277
	Hospitalization	82	88	85	328
	Overall	83	83	83	
	C.I.	81.3–85.3	81.1–85.2	81.0–85.2	
Early fusion	Non hospitalization	83	82	82	277
	Hospitalization	85	86	85	328
	Overall	84	84	84	
	C.I.	82.1–86.1	82.1–86.1	82.1–86.1	
Middle fusion	Non hospitalization	82	78	80	277
	Hospitalization	82	86	84	328
	Overall	82	82	82	
	C.I.	79.9–84.0	79.8–84.0	79.8–83.9	
Late fusion – w/o ‘history’ interval	Non hospitalization	76	75	75	277
	Hospitalization	79	80	79	328
	Overall	78	78	78	
	C.I.	76.5–79.8	75.6–79.7	75.5–79.7	
Early fusion – w/o ‘history’ interval	Non hospitalization	75	79	77	277
	Hospitalization	82	77	79	328
	Overall	78	78	78	
	C.I.	76.3–80.5	76.0–80.2	76.1–80.3	
Middle fusion – w/o ‘history’ interval	Non hospitalization	69	83	76	277
	Hospitalization	83	69	75	328
	Overall	77	75	75	
	C.I.	74.7–79.0	73.2–77.6	73.2–77.6	

Our EMR dataset is divided into ‘current’—15 days before COVID-19 test, and ‘history’ interval (data from 1 year before the test, excluding 15 days in the current history). It is evident that information from ‘history’ interval is crucial for future hospitalization prediction as the performance of fusion models without ‘history’ interval drops by an average of 6 F1-score (±1 std) than that of models with both ‘current’ and ‘history’ intervals.

**Fig. 2:**
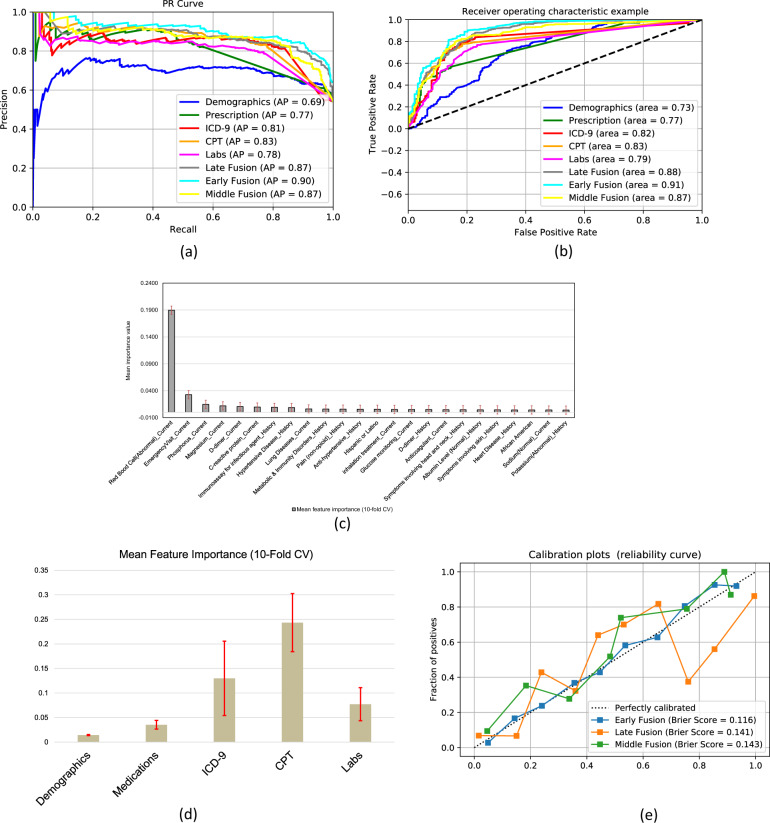
Statistical analysis of the models. **a** PR (left) and ROC (right) curves for model distinguishing between self-isolation and hospitalization outcomes. Each colored line represents a separate model and the color scheme is consistent between PR and ROC curves. Feature importance from (**b**) early fusion—shows the importance of top 25 individual EMR data component, **c** late Fusion model—shows the importance of individual EMR data sources. The standard deviation bar (red) is generated via 10-fold cross-validation on the training data. **d** Calibration curve for early, late and middle fusion models along with Brier scores for each calibrated model.

The receiver operating characteristics (ROC) curve and precision-recall (PR) curve are shown in Fig. 2a. Early (AUROC 0.91 & AUPRC 0.9), late (AUROC 0.88 & AUPRC 0.87) and middle (AUROC 0.87 & AUCPR 0.87) fusion achieve much higher Area under the Receiver Operating Curve (AUROC) and Area under the precision-recall curve (AUCPR), as compared to individual source classifiers. Interestingly, models trained on comorbidities coded as ICD9/10 and procedures performed on the patients also presents high performance.

We also performed calibration analysis of the three fusion models. Figure 2d shows calibration curves along with Brier scores for each model after calibration through isotonic regression. The early fusion model not only performs the best, but is the most reliable model with the lowest Brier score after calibration. While calibrated middle fusion tends to underestimate the positive class (risk of hospitalization), late fusion model seems to swing between over and under estimation with strong over estimation in the upper quadrant.

**Fig. 3:**
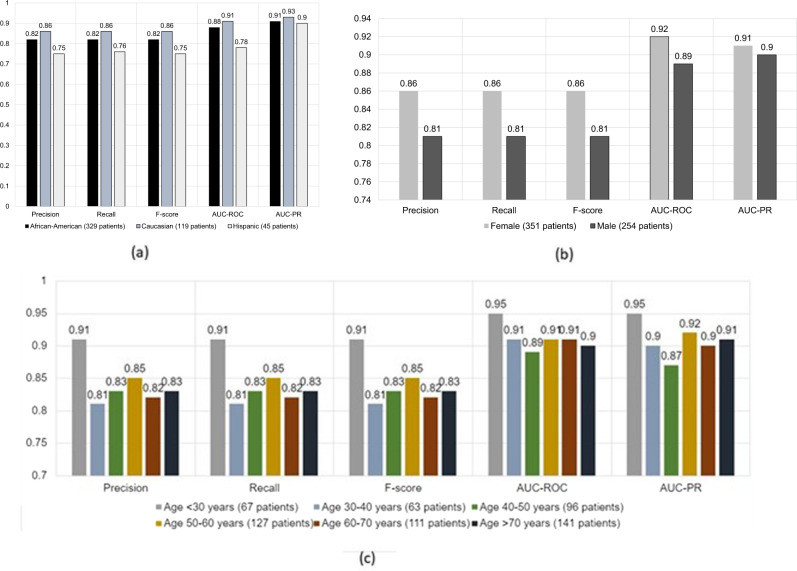
Performance stratification of the best performaing model based on Early fusion. **a** stratification based on race and ethnicity, **b** stratification based on gender, **c** stratification based on age.

We present the performance of the early fusion model stratified by race and ethnicity, gender, and age in Fig. 3a–c, respectively. In terms of race and ethnicity, the model performs equally well for all patients with a small drop in performance for Hispanic population which is probably bias given the smaller number of evaluation samples (see Supplementary Note 4 for detail). A similar performance drop is observed for male patients. In terms of age, our model achieves balance between most of the age ranges except for less-than-30-years category where the model achieves better performance. Generally healthier disposition of these patients may account for this performance difference.

**Fig. 4:**
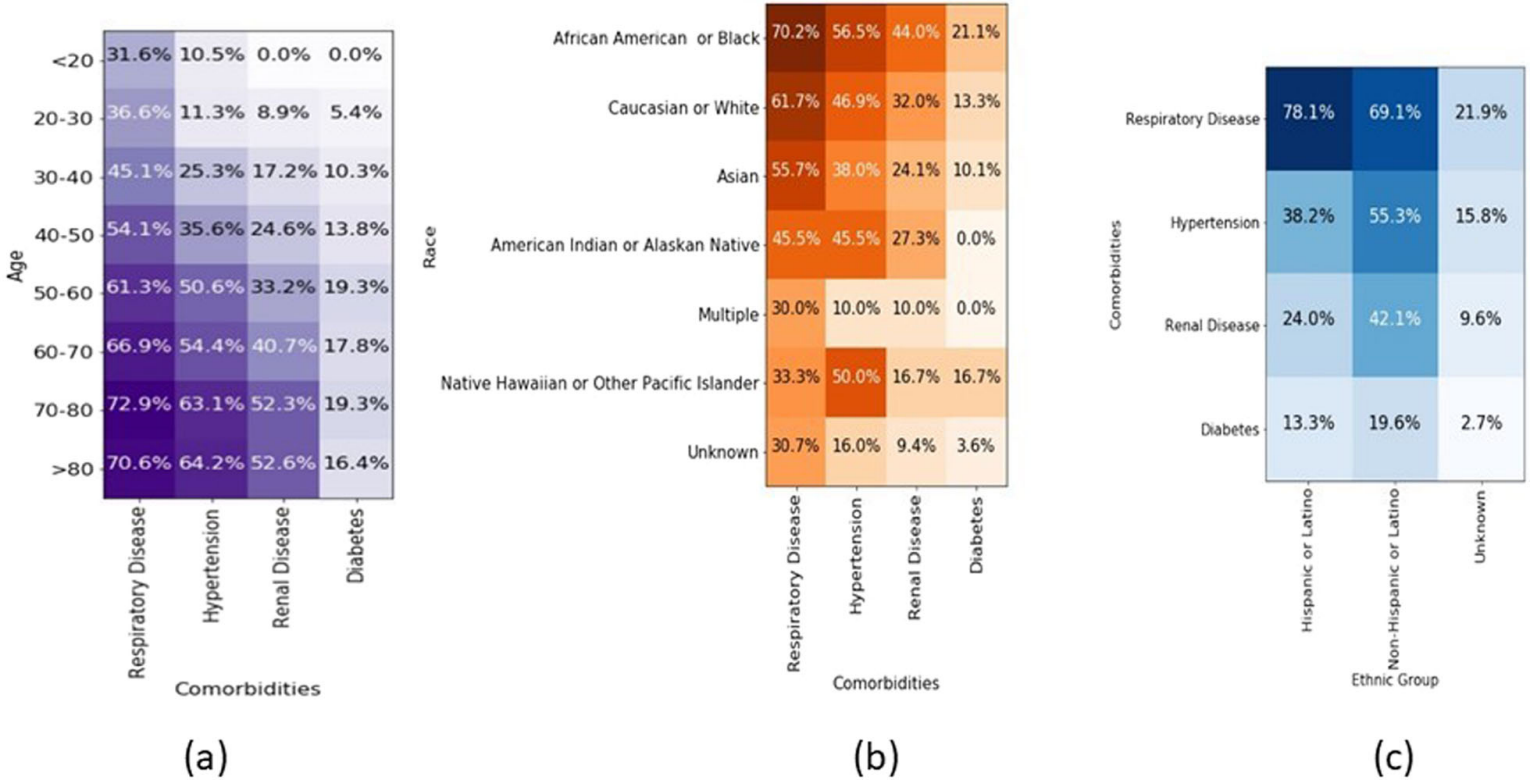
Patients characteristics as heatmaps. Heatmaps of **a** common comorbidities in our patient population according to different age groups, **b** relation between race and comorbidities, **c** relation between ethnic group and comorbidities. The value represented as % and darker color represents higher value.

**Fig. 5:**
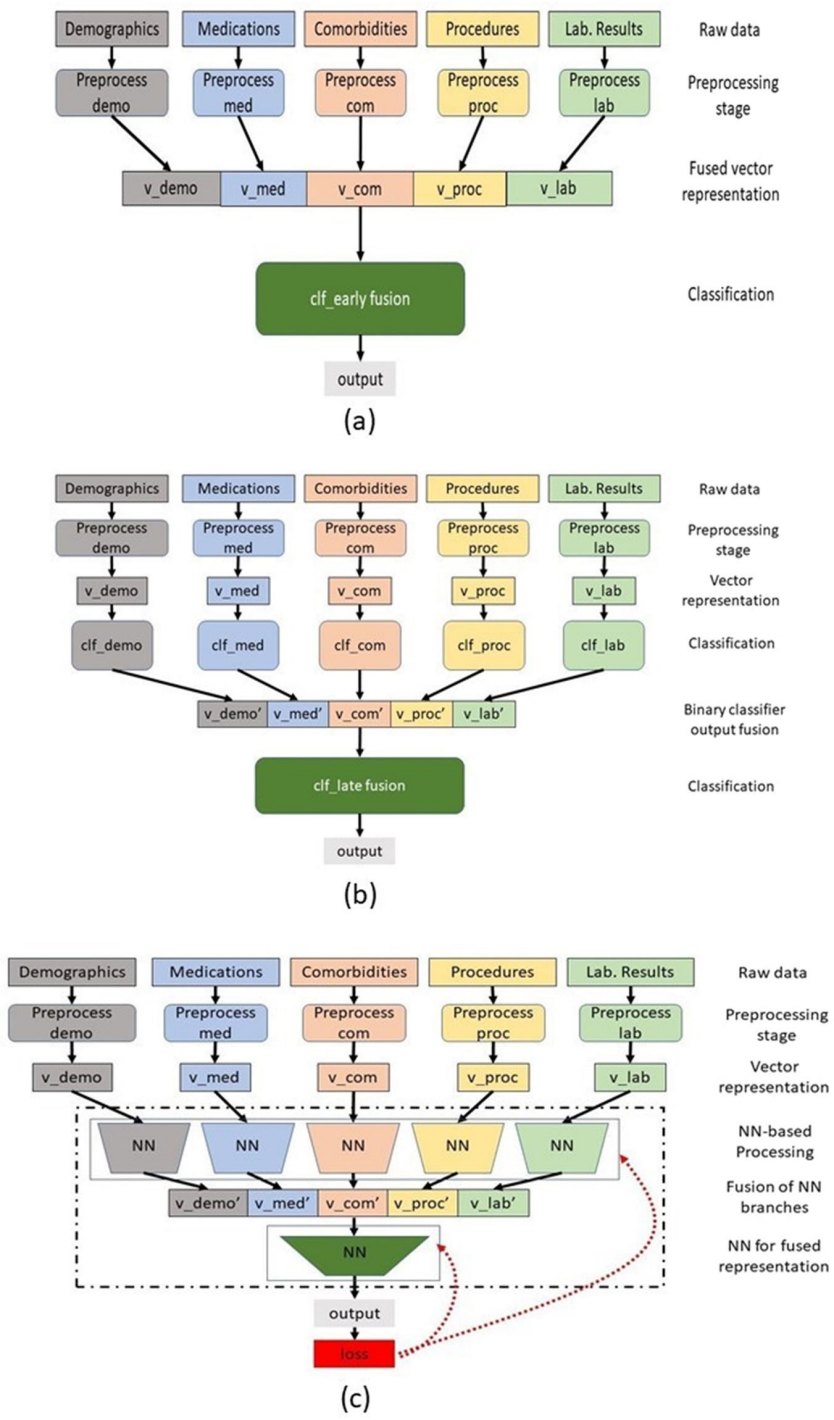
Proposed fusion AI model architectures. **a** Early fusion, **b** late fusion, **c** middle fusion/branched NN model.

### Feature importance

We investigated the interpretability of our best performing models, i.e., early and late fusion models, in terms of feature importance assigned to input features. The top features are shown as bar plots in Fig. 2b (early fusion) and Fig. 2c (late fusion) where we used 10-fold cross validation to compute average feature weights; standard deviation is shown as error bars. From the early fusion model, abnormal red blood cell counts, D-dimer test, history of hypertensive disease and previous emergency room encounters are most informative to predict hospitalization for patients with COVID-19. Demographic factors such as race and ethnicity (Black and Hispanic) as well as being male has high importance in prediction. Following the similar trend of the early fusion model, individual prediction using CPT and ICD data had higher weights in the late fusion meta-learner. Individual source model feature importance is presented in the Supplementary Note 4 and is consistent with the literature^3,16–18^: (1) comorbidities related to the lungs and urinary systems seem to be important for the classifier based on comorbidities, (2) treatment of thyroid-related diseases are given the highest importance by medications-based classifier.

## Discussion

In this study, we developed a multimodal fusion AI model from demographics, medications, laboratory tests, CPT, and ICD codes documented in the EMR to predict the severity of COVID-19 at the time of testing, and whether a COVID-19 patient will need hospitalization within 7 days of the RT-PCR test. This is in contrast to existing COVID-19 prediction models that employ medical information at the time of presentation to the hospital and predict an event between 24 h and 7 days into the future^5,6,8,9,19,20^. Our models rely on past health records of patients one year prior to testing. This enables our model to provide input to a dashboard that forecasts the utilization of hospital and ICU beds at the time of COVID-19 testing. As national efforts for testing scale up such a model can be used to further assign the patients the level of monitoring they will need based on their risk of disease progression. As mentioned in^10^, predictive models should serve a clinical need and use representative patients’ set. We have been careful to achieve both goals. We have used RT-PCR testing as a criterion to select a representative set of patients for COVID-19. Our model serves the clinical need of healthcare resource demand projection.

From a technical perspective, existing predictive models include logistic regression^4,12,21^, Lasso^13,19^, XGBoost^5^, Random Forest^6,8^, convolutional neural network^22^, semantic word embedding models^20,22^. We experimented with various classification models and found XGBoost and multi-branched deep dense network to be the most suitable. The technical novelty lies in a thorough exploration of vast and heterogeneous feature spaces, handling of information collected over long time periods, and their intuitive fusion with minimal expert supervision, Lasso^13,19^, XGBoost^5^, Random Forest^6,8^, convolutional neural network^22^, semantic word embedding models^20,22^. We experimented with various classification models and found XGBoost and multi-branched deep dense network to be the most suitable. The technical novelty lies in a thorough exploration of vast and heterogeneous feature spaces, handling of information collected over long time periods, and their intuitive fusion with minimal expert supervision.

A review of feature importance provides insight for future research and feedback from the community on the significance of various predictors of COVID-19 disease trajectory. For example, several papers have been published on the disparate outcome based on race and ethnicity, with more deaths observed in blacks and Hispanics^23,24^. When only demographics are used in the model, they have a lower F1 score (69% versus 84% for the early fusion model), which could potentially be explained by other clinical and systemic factors that contribute to worse severity among minorities. In our sample cohort, the distribution of comorbidities (respiratory illness, hypertension, renal disease and diabetes) is weighted heavily among African Americans and older patients (>50 years). This may explain the weighting of the CPT and ICD codes that represent interventions and patient comorbidities. Overall model performance does not vary across races and ethnicity. A review of feature importance provides insight for future research and feedback from the community on the significance of various predictors of COVID-19 disease trajectory. For example, several papers have been published on the disparate outcome based on race and ethnicity, with more deaths observed in blacks and Hispanics^23,24^. When only demographics are used in the model, they have a lower F1 score (69% versus 84% for the early fusion model), which could potentially be explained by other clinical and systemic factors that contribute to worse severity among minorities. In our sample cohort, the distribution of comorbidities (respiratory illness, hypertension, renal disease, and diabetes) is weighted heavily among African Americans and older patients (>50 years). This may explain the weighting of the CPT and ICD codes that represent interventions and patient comorbidities. Overall model performance does not vary across races and ethnicity.

Inflammatory marker laboratory levels like procalcitonin, ferritin, and lactate noted to be important for COVID-19 care are not routinely collected in care, and hence are not represented in the top laboratory markers in our patient cohort. Our models show that the immediate pre-testing period is an important predictor of COVID-19 severity and need for hospitalization, especially when patients are recently started on anticoagulation, thyroid, or respiratory medications. Moreover, the complete blood count has the highest feature importance. To our knowledge, the complete blood count has not been linked to COVID-19 disease course.

Our study has important limitations. The models were trained on a population of patients who were cared for in a highly integrated academic healthcare system with 56.4% African American and 2% Asian population. The models may not perform well in a different patient demographic or health system. Second, the number of patients for training and validation is limited given we only consider patients with RT-PCR tests before September 2020. The limited number of patients with sparse data make the modeling problem challenging. Even though early fusion results in the best prediction, statistical metrics (precision, F1-score) indicate late and middle fusion results are very similar (*p* < 0.05, see Supplementary Note 4). We believe that middle fusion with consistent backpropagation may generate the optimal result with larger training data.

## Methods

### Cohort description

With the approval of Emory Institutional Review Board (IRB), we collected all the EMR data from all patients flagged as COVID-19 positive (ICD10 diagnosis code - U07.1 + codes for symptoms or notes in the record) in 12 different facilities in Emory University Healthcare (EUH). Since only de-identified data were used, IRB waived off the requirement of informed consent by the patients. Between January and September 2020, there were 3194 such patients. We collected PCR testing information available from all EUH facilities. We found that 3120 of 3194 patients had at least one positive PCR test for COVID-19. The remaining patients either had no positive test or had missing test results. We collected all hospitalization (admission/discharge) data for COVID-19 positive patients from January 2020. We carefully examined the data to identify patients who were admitted to the hospital after testing positive for COVID-19, but excluding hospitalization unrelated to COVID-19 (i.e., hospitalization after 7 days of RT-PCR testing).

Figure 1a shows the overall architecture of our model including possible outcomes. Figure 1b shows inclusion and exclusion criteria for selecting patients that were hospitalized or not hospitalized after COVID-19 testing. We found 1504 patients who were hospitalized with COVID-19 diagnosis and 1340 patients who were not hospitalized. The rest had irreconcilable information including hospitalization before testing positive for COVID-19. Such hospitalization may be unrelated to COVID-19 or the patient self-quarantined early after testing but later had to be admitted to hospital (more than 7 days after testing), indicating progression of the disease. Of the 1504 patients admitted to the hospital, 365 patients were later admitted to ICU while the remainder stayed in a regular inpatient ward.

Table 2 highlights the overall characteristics of our patient populations, including comorbidities, and Fig. 4a–c shows the common comorbidities in our patient population for different age groups and the correlation between race, ethnicity, and comorbidities.

We aim to develop an AI model to help plan healthcare resource needs for each COVID-19 patient by predicting the need for hospitalization at the time the patient takes a RT-PCR test (Fig. 1a). Our predictive models employ retrospective EMR data prior to COVID-19 testing, including diagnoses, prescribed medications, laboratory test results, and demographics collected over a year prior to the test. In our dataset, such information is available from January 2019 to September 2020. Our study complies with TRIPOD^25^ guidelines for reporting. Our study complies with TRIPOD^25^ guidelines for reporting. Cohort and models are described in the following sub-sections. Performance is reported in the Results section and interpretation of results and limitations of our approach are detailed in the “Discussion” section.

### Handling temporal EMR data

Since the clinical encounter data have been generated over more than a year time-period, it is important for the model to be able to differentiate and put justifiable emphasis over more recent versus historical medical information. However, the COVID-19 pandemic resulted in a scenario where patients may have their first healthcare encounter due to infection with very little past medical history. Therefore, the generated EMR data are very sparse and finer time-interval division results in prohibitively large fraction of missing data values. To handle such missing data and at the same time achieve temporal distinction between information, we divide EMR data for each patient into two intervals, i.e., *current* and *history* (Fig. 1a). The *current* interval includes all information collected between 24 h before the RT-PCR test and 15 days before the time of test. The history interval includes all information collected prior to the current interval. We experimented with several temporal data splitting schemes including weekly, monthly, and quarterly splits. The sparsity of data renders most of these splits suboptimal for modeling. We observed that above mentioned scheme of current and history interval suffices for distinguishing between EMR information on the temporal axis for the given problem while avoiding insurmountable data sparsity.

### Multi-modal EMR data

The following data were extracted from the EMR.

1. *Demographic information:* includes gender (male/female), race (African American, Caucasian, Native Hawaiian or Other Pacific Islander, Asian, American Indian or Alaska Native, Multiple, Unknown), ethnic group (Hispanic or Latino, Non-Hispanic or Latino, Unknown), and age in years.
2. *In-patient and out-patient medications:* With physician feedback and RxNorm categorization, we created groups for important medications of the top 50 most-frequent in-patient and out-patient medications into 21 distinct groups. Details of medication and medication groups are provided in Supplementary Note 2. If a certain medication is not mentioned in a patient’s record, it is assumed that the patient was not prescribed or administered that medication.
3. *CPT code:* We selected all CPT codes occurring at least 500 times in the dataset, resulting in a set of 168 features. If a code is not mentioned in a patient’s record, we assume that the procedure corresponding to that CPT code was never performed for the patient.
4. *Comorbidities*: are coded as ICD-9 codes which we grouped based on hierarchical structure^26^. Further details are provided in the supplementary material. We use each group as a feature resulting in 108 distinct features.
5. *Comorbidities*: are coded as ICD-9 codes which we grouped based on hierarchical structure^26^. Further details are provided in Supplementary Note 2. We use each group as a feature resulting in 108 distinct features.
6. *Laboratory test results:* included in our data are coded in Logical Observation Identifiers Names and Codes (LOINC). We selected 30 most frequent laboratory tests. Each laboratory test value for a patient is coded as ‘Normal’ (value within normal range), ‘Abnormal’ (value outside of normal range), and ‘Unknown’ (no value provided). Selected laboratory tests and their normal ranges are provided in Supplementary Note 2.

For each modality except for demographics which remains unchanged between current and history interval, feature values from each interval were concatenated to form a representation vector.

### Fusion AI models development

In order to integrate data from different EMR sources, we explored three types of fusion techniques—early, late and middle fusion^14^ combined with various classification models including Logistic Regression, Random Forest, Multi-layer neural network, and XG Boost^15^.

Figure 5 summarizes the proposed fusion architectures used in our methodology.

- *Early Fusion* is commonly known as ‘feature-level’ fusion where we concatenated features from all selected sources in a single vector representation that is passed as input through an AI model. Chao et al. used an early fusion type model to combine information from lung imaging data with demographic information, blood tests, and vitals to predict ICU admission^27^. We have experimented with early fusion to combine a wider variety of non-imaging information including demographic features, CPT and ICD-9/10 codes, laboratory test results and past medications. The core challenge is that the EMR feature values are highly heterogeneous, and include categorical, continuous, and text representation. They also need to be normalized before concatenation. In our dataset, all demographic features except for age are categorical resulting in 0/1 feature values. We normalized the continuous feature of age such that its value lies between 0 and 1. Medication and CPT codes are nominal features normalized between 0 and 1. Comorbidities (ICD-9 groups) are categorical features with 0/1 values. Each lab results are formulated in three categorical features, i.e., ‘Normal’, ‘Abnormal’, and ‘Unknown’. We experimented with four discriminative models (Logistic Regression, Random Forest, Multi-layer Perceptron, XG Boost^15^) for early fusion once concatenated feature vector was generated. We experimented with four discriminative models (Logistic Regression, Random Forest, Multi-layer Perceptron, XG Boost^15^) for early fusion once concatenated feature vector was generated.
- Late Fusion is known as ‘decision-level’ fusion where feature vectors from each modality are passed through separate discriminative models and result probability values are concatenated to form a final feature vector for each patient. For example, Ning et al.^28^ used deep learning-based late fusion architecture to create feature vectors based on probabilities estimated by processing CT and CF data for COVID-19 patients through CNN and DNN, respectively. This feature vector is passed through a meta-learner to combine the prediction of each model and generate the final label. The meta-learner is trained to learn the importance of each prediction source, instead of each individual features, and the most predictive source (source with highest prediction accuracy) is expected to weigh high in the meta-learner. In our experiments, XGBoost was the best performing discriminator for demographics, medications, and comorbidities while Random Forest was the most accurate discriminator for CPT codes and laboratory test results. We used XGBoost as meta-learner based on its performance. These selections were made by experimenting with the training set.
- *Middle Fusion/Branched NN model* joins the learned feature representation from intermediate layers of the neural network with the features from other sources. We designed a branched neural network (NN) model for the middle fusion technique. Feature vector from reach modality is passed through a separate branch of NN model consisting of dense, dropout, and activation layers. The resulting compressed representation from each branch are concatenated and passed through another branch consisting of dense, dropout and activation layer, to generate the final output (Fig. 5c). We performed detailed hyperparameter tuning to determine the optimal number of layers in each branch, dropout rate, activation, number of epochs, and optimizer (see Supplementary Note 3).

In addition to the fusion models, we developed machine learning models using each EMR source individually to examine the importance of that information source for prediction. We randomly select 80% (2275 patients) of the total cohort to train the models and evaluate the performance on the rest (20%, 569 patients). The same test set is used to validate all the models and the training set was further divided to tune the hyperparameters.

### Reporting summary

Further information on research design is available in the Nature Research Reporting Summary linked to this article.

## Supplementary information

Supplementary Information

Reporting Summary

## Data Availability

The datasets generated and analyzed during the study are not currently publicly available due to HIPAA compliance agreement but are available from the corresponding author on reasonable request with proper data usage agreement.

## Code availability

Code will be made public after the publication of this work at https://github.com/ATARIQ5
