# Supplementary material for: Patient-specific COVID-19 resource utilization prediction using fusion AI model
Source: NPJ Digit Med. 2021 Jun 3;4:94. doi: 10.1038/s41746-021-00461-0 (PMC8175333; doi:10.1038/s41746-021-00461-0)
Supplement: Supplementary file 1 — Supplementary Information [file 41746_2021_461_MOESM1_ESM.pdf]

Supplementary Material

Contents

Supplementary Note 1: Prior work 1
Supplementary Note 2: Feature Engineering: 2
Comorbidities: 2
Medications: 6
Laboratory Results: 8
CPT Codes: 9
Supplementary Note 3: Modeling 9
Classification Models: 9
Hyperparameter Tuning for Middle Fusion: 10
Supplementary Note 4: Performance Evaluation: 12
Statistical Significance of Differences in Fusion Models: 12
Race and ethnicity-wise performance: 13
Feature importance for individual EMR modalities: 17
Supplementary References: 21

Supplementary Note 1: Prior work

Supplementary Table 1 is a list of published papers regarding COVID-19 related clinical event prediction. Most of these publications do not focus on the full EMR of patients, and only rely on a manual selection of limited features. There is also universal lack experimentation on fusion and temporal modeling. We have listed the limitations of each work in the Table 1.

Supplementary Table 1: List of prior prediction works on COVID-19

Citations	Limitations
[1]	Logistic regression model, Multiple EMR modalities.<br>Cons: Fusion methodologies and discriminative models were not properly evaluated. Only experimented with Early fusion.
[2]	Uses CNN and word2vec<br>Cons: analysis of only the textual part of EMR data
[3]	BERT for symptom prediction<br>Cons: symptom-disease relation analysis, no direct future clinical event prediction
[4]	Simple 2-layer denser network

	Cons: 40 manually selected features are directly fed as input, not focused on fusion, temporal modeling or automated feature selection
[5]	Uses Random Forest on multi-modal EMR data<br>Cons: Features are manually selected by experts, no proper validation of fusion or temporal modeling
[6]	Mortality prediction, uses logistic regression<br>Cons: Very simple modeling through logistic regression, no experimentation with fusion
[7]	Survival calculation, LASSO modeling<br>Cons: no experimentation with fusion
[8]	Uses XGBoost, Prediction of mortality and other clinical events, Multi-center data (trained on data from one center, evaluated on data from another center)<br>Cons: Manual feature selection, No experimentation with fusion
[9]	Near-term hospitalization prediction, Uses Random Forest classifier<br>Cons: Manual feature selection based on past literature, only immediate hospitalization is predicted, No experimentation with fusion
[10]	Uses LASSE and multi-layer perceptron, 'federated' leaning indicates averaging out of parameter weights from models trained on different sites<br>Cons: no experimentation with fusion and feature selection
[11]	Predicts score to indicate future hospitalization, critical illness, or death, large cohort<br>Cons: Manually selected predictors, no experimentation with fusion or temporal modeling
[12]	Involves chest X-ray with other EMR data<br>Cons: manual inspection of X-rays, manual feature selection, no thorough experimentation with fusion or temporal modeling

Supplementary Note 2: Feature Engineering:

Comorbidities:

Supplementary Table 2 shows ICD-9 code groups and subgroups. We used each subgroup as a feature. This grouping is based on hierarchical structure of ICD-9 codes [13] (see Table 2).

Supplementary Table 2: ICD9 grouping strategy

ICD-9 Start	ICD-9 End	Group Description	Subgroup Description
760	763.99	Certain conditions originating in the perinatal period	Maternal causes of perinatal morbidity and mortality
764	779.99		Other conditions originating in the perinatal period
640	640.99	Complications of pregnancy, childbirth, and the puerperium	Complications mainly related to pregnancy
660	669.99		Complications occurring mainly in the course of labor and delivery
670	677.99		Complications of the puerperium

630	633.99		Ectopic and molar pregnancy
650	659.99		Normal delivery, and other indications for care in pregnancy, labor, and delivery
678	679.99		Other maternal and fetal complications
634	639.99		Other pregnancy with abortive outcome
740	740		Anencephalus and similar anomalies
748	748		Anomalies of respiratory system, congenital
745	745		Bulbus cordis anomalies and anomalies of cardiac septal closure
754	754		Certain congenital musculoskeletal deformities
758	758		Chromosomal anomalies
749	749		Cleft palate and cleft lip
744	744		Congenital anomalies of ear, face, and neck
743	743		Congenital anomalies of eye
752	752		Congenital anomalies of genital organs
757	757		Congenital anomalies of the integument
753	753		Congenital anomalies of urinary system
759	759		Other and unspecified congenital anomalies
747	747		Other congenital anomalies of circulatory system
751	751		Other congenital anomalies of digestive system
746	746		Other congenital anomalies of heart
755	755		Other congenital anomalies of limbs
742	742		Other congenital anomalies of nervous system
750	750		Other congenital anomalies of upper alimentary tract
756	756		Other congenital musculoskeletal anomalies
741	741		Spina bifida
283	283	Congenital anomalies	Acquired hemolytic anemias
284	284		Aplastic anemia and other bone marrow failure syndromes
286	286		Coagulation defects
288	288		Diseases of white blood cells
282	282		Hereditary hemolytic anemias
280	280		Iron deficiency anemias
285	285		Other and unspecified anemias
281	281		Other deficiency anemias
289	289		Other diseases of blood and blood-forming organs
287	287		Purpura and other hemorrhagic conditions
390	392.99	Diseases of the blood and the blood forming organs	Acute rheumatic fever
430	438.99		Cerebrovascular disease
393	398.99		Chronic rheumatic heart disease
440	449.99		Diseases of arteries, arterioles, and capillaries
415	417.99		Diseases of pulmonary circulation
451	459.99		Diseases of veins and lymphatics, and other diseases of circulatory system
401	405.99		Hypertensive disease
410	414.99	Diseases of the circulatory system	Ischemic heart disease

420	429.99		Other forms of heart disease
540	543.99	Diseases of the digestive system	Appendicitis
530	539.99		Diseases of esophagus, stomach, and duodenum
520	529.99		Diseases of oral cavity, salivary glands, and jaws
538	538		Gastrointestinal mucositis (ulcerative)
550	553.99		Hernia of abdominal cavity
555	558.99		Noninfectious enteritis and colitis
570	579.99		Other diseases of digestive system
560	569.99		Other diseases of intestines and peritoneum
600	608.99	Diseases of the genitourinary system	Diseases of male genital organs
610	612.99		Disorders of breast
614	616.99		Inflammatory disease of female pelvic organs
580	589.99		Nephritis, nephrotic syndrome, and nephrosis
590	599.99		Other diseases of urinary system
617	629.99		Other disorders of female genital tract
710	719.99	Diseases of the musculoskeletal system and connective tissue	Arthropathies and related disorders
720	724.99		Dorsopathies
730	739.99		Osteopathies, chondropathies, and acquired musculoskeletal deformities
725	729.99		Rheumatism, excluding the back
380	389.99	Diseases of the nervous system and sense organs	Diseases of the ear and mastoid process
360	379.99		Disorders of the eye and adnexa
350	359.99		Disorders of the peripheral nervous system
330	337.99		Hereditary and degenerative diseases of the central nervous system
320	326.99		Inflammatory diseases of the central nervous system
327	327.99		Organic sleep disorders
340	349.99		Other disorders of the central nervous system
339	339.99		Other headache syndromes
338	338.99		Pain
460	466.99		Acute respiratory infections
490	496.99	Diseases of the respiratory system	Chronic obstructive pulmonary disease and allied conditions
510	519.99		Other diseases of respiratory system
470	478.99		Other diseases of the upper respiratory tract
500	508.99		Pneumoconiosis and other
480	488.99		Lung diseases due to external agents
680	686.99		Pneumonia and influenza
700	709.99	Diseases of the skin and subcutaneous tissue	Other diseases of skin and subcutaneous tissue
690	698.99		Other inflammatory conditions of skin and subcutaneous tissue
249	259.99	Endocrine, nutritional and metabolic diseases, and immunity disorders	Diseases of other endocrine glands
240	246.99		Disorders of thyroid gland
260	269.99		Nutritional deficiencies
270	279.99		Other metabolic and immunity disorders
60	66.99	Infectious and parasitic diseases	Arthropod-borne viral diseases
120	129.99		Helminthiases

42	42.99		Human immunodeficiency
1	9.99		Virus [HIV] infection
137	139.99		Intestinal infectious diseases
110	118.99		Late effects of infectious and parasitic diseases
30	41.99		Mycoses
70	79.99		Other bacterial diseases
130	136.99		Other diseases due to viruses and chlamydia
100	104.99		Other infectious and parasitic diseases
45	49.99		Other spirochetal diseases
80	88.99		Poliomyelitis and other non-arthropod-borne viral diseases and prion diseases of central nervous system
90	99.99		Rickettsioses and other arthropod-borne diseases
10	18.99		Syphilis and other venereal diseases
50	59.99		Tuberculosis
20	27.99		Viral diseases generally accompanied by exanthem
940	949.99	Injury and poisoning	Burns
958	959.99		Certain traumatic complications and unspecified injuries
996	999.99		Complications of surgical and medical care, not elsewhere classified
920	924.99		Contusion with intact skin surface
925	929.99		Crushing injury
830	839.99		Dislocation
930	939.99		Effects of foreign body
800	829.99		Entering through orifice fractures
900	904.99		Injury to blood vessels
950	957.99		Injury to nerves and spinal cord
860	869.99		Internal injury of thorax, abdomen, and pelvis
850	854.99		Intracranial injury, excluding those with skull fracture
905	909.99		Late effects of injuries, poisonings, toxic effects, and other external causes
870	897.99		Open wounds
990	995.99		Other and unspecified effects of external causes
960	979.99		Poisoning by drugs, medicinal and biological substances
840	848.99		Sprains and strains of joints and adjacent muscles
910	919.99		Superficial injury
980	989.99		Toxic effects of substances chiefly nonmedicinal as to source
317	319.99		Intellectual disabilities
300	316.99	Mental, behavioral and neurodevelopmental disorders	Neurotic disorders, personality disorders, and other nonpsychotic mental disorders
290	299.99		Psychoses
140	239.99	Neoplasms	Neoplasms
797	799.99		Ill-defined and unknown causes of morbidity and mortality
790	796.99	Symptoms, signs, and ill-defined conditions	Nonspecific abnormal findings
780	780		General symptoms
789	789		Other symptoms involving abdomen and pelvis

783	783		Symptoms concerning nutrition, metabolism, and development
785	785		Symptoms involving cardiovascular system
787	787		Symptoms involving digestive system
784	784		Symptoms involving head and neck
781	781		Symptoms involving nervous and musculoskeletal systems
786	786		Symptoms involving respiratory system and other chest symptoms
782	782		Symptoms involving skin and other integumentary tissue
788	788		Symptoms involving urinary system
E000	E999.99	Symptoms, signs, and ill-defined conditions	Supplementary classification of external causes of injury and poisoning
V01	V91.99	Supplementary classification of factors influencing health status and contact with health services	Supplementary classification of factors influencing health status and contact with health services

Medications: Supplementary Table 3 shows medication groups used as features. The idea behind this grouping is to enhance meaningfulness of features for the task of outcome prediction. A patient may have been prescribed or administered more than one medication from a group. In such cases, feature value is the number of medications prescribed from the given medication group. Thus, feature values are integers greater than or equal to 0. We employed MinMax scaler to normalize the values to lie between 0 and 1.

Supplementary Table 3: Grouping of medications

**Medication**	**Medication Group**
propofol	
dexmedetomidine	
ketamine	
vecuronium	Anesthesia
vancomycin	
azithromycin	
doxycycline	
sulfamethoxazole-trimethoprim	Antibiotic
enoxaparin	
bivalirudin	
heparin	
argatroban	
apixaban	Anticoagulant

clopidogrel	
gabapentin	
pregabalin	Anti-epileptic
hydrALAZINE	
niCARdipine	
amLODIPine	
hydroCHLOROthiazide	
lisinopril	
losartan	
torsemide	Anti-hypertensive
midazolam	
traZODone	Anxiolytic
carvedilol	
metoprolol	
amiodarone	Cardiovascular
metFORMIN	Diabetes
furosemide	Diuretic
magnesium sulfate	
aspirin	
calcium gluconate	Electrolyte
Lactated Ringers Injection intravenous solution	
Sodium Chloride 0.9% intravenous solution	
potassium chloride	
Dextrose 5% in Water intravenous solution	
Premix NS	
sterile water	
Electrolyte (Plasma-Lyte) intravenous solution	
Premix Dextrose 5%	Fluid
insulin glargine	
insulin lispro	
insulin regular	Insulin
acetaminophen	
cyclobenzaprine	
diclofenac topical	
ibuprofen	Pain (non-opioid)
HYDROmorphone	
fentaNYL	
oxyCODONE	
acetaminophen-hydrocodone	
acetaminophen-oxycodone	
traMADol	Pain (opioid)
norepinephrine	
vasopressin	Pressor

albuterol-ipratropium	Respiratory
albuterol	
atorvastatin	Statin
pravastatin	
methylPREDNISolone	Steroid
predniSONE	
levothyroxine	Thyroid
milrinone	Vasodilator

Laboratory Results:

Supplementary Table 4 presents the 30 most frequent laboratory tests in our dataset, along with the upper and lower thresholds used for labeling as 'Normal' or 'Abnormal'. All of these laboratory tests were grouped into feature values as 'Normal', 'Abnormal', or 'Unknown'.

Supplementary Table 4: Value range for the laboratory results

**Structured Result Type**	**Lower Value**	**Upper Value**
AG	3	10
White Blood Count	4	11
Hemoglobin	12	17.5
Hematocrit	38.5	45.5
Red Blood Cell Count	4.35	5.65
Red Cell Distribution Width-CV	11.8	16.1
Auto Nucleated Red Blood Cell, Absolute	0.3	339
Auto Nucleated Red Cell Count	1	390
Mean Platelet Volume	9.4	12.3
MCHC	33.4	35.5
MCH	27.5	33.2
MCV	80	100
Platelet count	150	450
Estimated GFR, African American	72.6	108.9
Estimated GFR, Non-African American	60	90
Glucose	70	80
Potassium	3.6	5.2
Chloride	96	106
Calcium	8.6	10.3
Creatinine	0.84	1.21
Sodium	135	145
Blood Urea Nitrogen	7	20

Osmolality, Calculated	285	295
Carbon Dioxide	23	29
Albumin	3.4	5.4
Aspartate Aminotransferase	7	37
Protein	6	8.3
Bilirubin	0.1	1.2
Alanine Aminotransferase	24	29
Alkaline Phosphatase	44	147

CPT Codes:

We applied frequency-based filtering for selecting CPT codes to be used as features. Once selected, we counted the number of times each CPT was mentioned for a patient. We believe it is important if a patient had certain procedure multiple times. Thus, feature values were integers greater than or equal to 0. We normalized the values to lie between 0 and 1 using MinMax scaler of SKLearn library.

Supplementary Note 3: Modeling

Classification Models:

We selected a vast set of classification models to include in our experiments. The reported results for each case (prediction based on individual EMR modality as well as fusion modeling) are based on the predictions of the best performing model in each case. Supplementary Table 5 shows the best performing model for every modality.

Supplementary Table 5: Best performing classifiers for individual modalities and fusion modeling

EMR Modality	Discriminator
Demographics	XGBoost
Medications	XGBoost
Comorbidities (ICD codes)	XGBoost
CPT codes	Random Forest
Laboratory Tests	Random Forest
Late Fusion	XGBoost

Early Fusion	XGBoost

Logistic regression is a simplistic model that we included to determine a baseline. Support Vector Machine (SVM) is known for high performance with low memory consumption, in high-dimensional features space. Since we included a large number of features as predictors, we selected SVM for experimentation. Random Forest (RF) is known for robustness to outliers and non-linearity in feature space. We included RF and XGBoost. Like RF, XGBoostis also based on decision trees and has shown better performance and higher execution speed on various problem through the use of gradient boosting. Neural Networks (NN) provides flexibility in terms of model design. We experimented with the number and sizes of layers as well as non-linearity to find a suitable design. For middle fusion, we used deep learning to design a customized branched network where each branch design is individually tuned for the EMR modality it corresponds to. Tuning details are provided in the following section.

Hyperparameter Tuning for Middle Fusion:

The structure of each branch in the model is proportional to the dimensionality of the corresponding EMR modality (demographics: 14, medication: 21 (no. of medication groups) x 2 (current, history), Comorbidities: 114 (no of ICD-9 sub-groups) x2 (current, history), CPT: 168 (no. of selected CPT codes) x 2 (current, history), Labs: 30 (no. of labs) x3 (normal/abnormal/unknown) x 2 (current, history)). We tuned the model over number of epochs (25, 50, 100), learning rates (1e-4, 1e-5, 1e-6), activations (tanh, relu, softmax), optimizers (adam, sgd, rmsprop), drop-out rate (0.2, 0.25, 0.5), as well as network size in terms of number of layers in each branch (large, small). The large network has 2, 4, 7, 7, 6, and 4 dense layers in demographics, medications, CPT, comorbidities, labs, and fused branches, respectively. All dense

layers (except for the last dense layer in each branch) are followed by activation and dropout layers. The small network has 1, 2, 4, 4, 4, and 3 dense layers in demographics, medications, CPT, comorbidities, labs, and fused branches, respectively. Supplementary Table 5 shows top-25 hyperparameter value combination in terms of weighted average F-score (sorted in descending order of F-score).

Supplementary Table 6: Top 25 hyperparameters listed with average f1-score

**Model**	**Activation**	**Learning Rate**	**Optimizer**	**Dropout Rate**	**Epochs**	**Weighted Avg. F-score**
Large	tanh	0.0001	rmsprop	0.2	100	82
Large	tanh	0.0001	adam	0.25	100	81
Small	tanh	0.0001	adam	0.2	50	81
Small	tanh	0.0001	adam	0.2	100	81
Large	tanh	0.0001	adam	0.2	100	80
Large	tanh	0.0001	rmsprop	0.2	50	80
Large	tanh	0.0001	rmsprop	0.25	50	80
Small	tanh	0.0001	rmsprop	0.2	25	80
Small	tanh	0.0001	rmsprop	0.2	50	80
Small	tanh	0.0001	rmsprop	0.2	100	80
Small	tanh	0.0001	rmsprop	0.25	100	80
Large	tanh	0.0001	adam	0.25	50	79
Large	tanh	0.0001	rmsprop	0.25	25	79
Small	tanh	0.0001	adam	0.25	100	79
Small	relu	0.0001	rmsprop	0.5	50	79
Large	tanh	0.0001	adam	0.2	50	78
Large	tanh	0.0001	rmsprop	0.2	25	78
Large	tanh	0.0001	rmsprop	0.25	100	78
Small	tanh	0.0001	adam	0.25	25	78
Small	tanh	0.0001	adam	0.25	50	78
Small	tanh	0.0001	rmsprop	0.25	50	78
Small	tanh	0.00001	rmsprop	0.25	100	78
Large	tanh	0.0001	adam	0.25	25	77
Large	tanh	0.00001	rmsprop	0.2	100	77
Large	relu	0.0001	rmsprop	0.2	100	77

Supplementary Note 4: Performance Evaluation:

Statistical Significance of Differences in Fusion Models:

As shown the Table 2 of the main manuscript, all three fusion models achieve very similar performance with early fusion gaining a slight edge. The following p-value matrix (Supplementary Figure 1) is the result of independent t-test between every pair of fusion models results. It clearly indicates that models' outputs are very similar to each other with their differences being statistically insignificant (statistical significance is indicates by $p<0.05$).

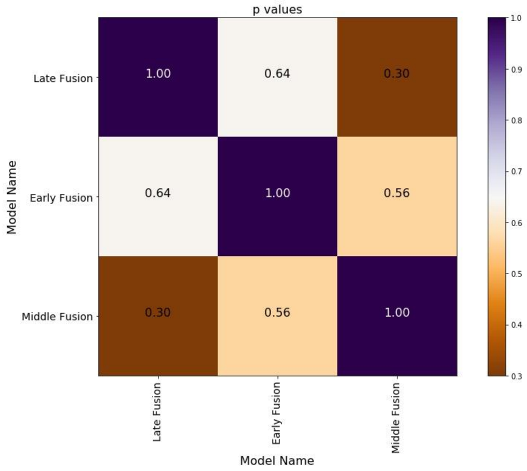

Supplementary Figure 1: p-values of independent t-test between all pairs of fusion models

Race and ethnicity-wise performance:

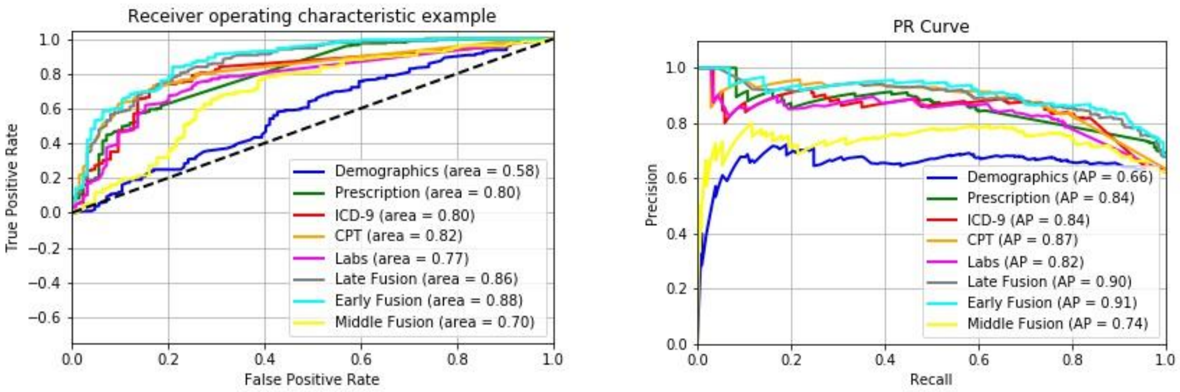

(a)

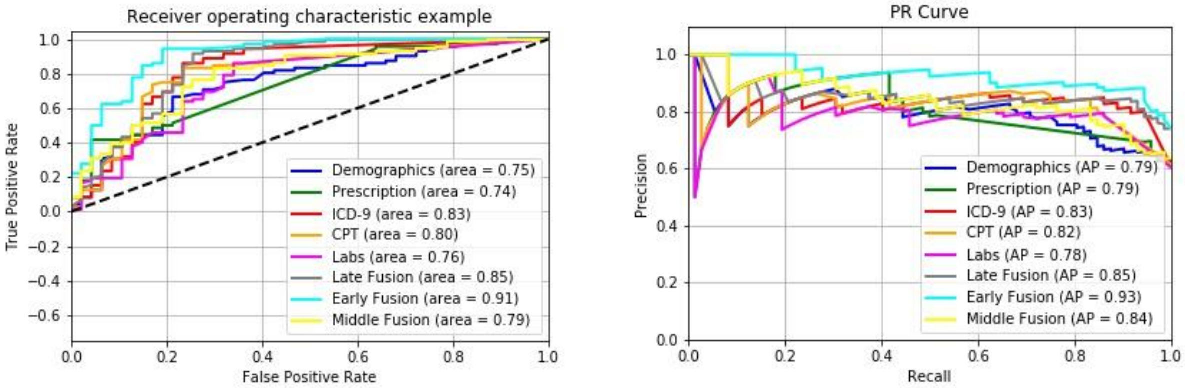

(b)

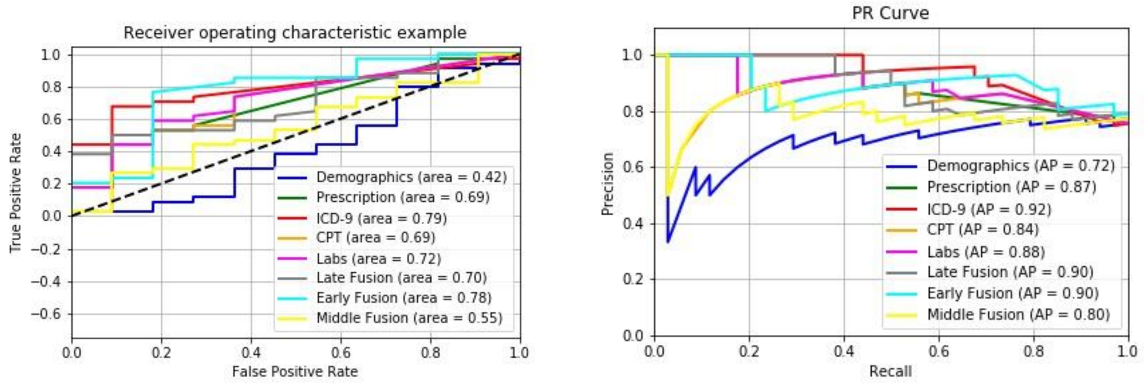

(c)

Supplementary Figure 2: Statistical analysis of the models on the African American population (a), Caucasian (b) and Hispanic (c) patients : (a) PR (left) and ROC (right) curves for model distinguishing between non hospitalization and hospitalization outcomes. Each colored line represents a separate model and the color scheme is consistent between PR and ROC curve.

Supplementary Table 7: Performance of the model on the African American population

		Precision	Recall	F-score	Support
Demographics	Non hospitalization	49	36	42	124
	Hospitalization	67	78	72	205
	Overall	60	62	61	
	C.I.	56.6-64.0	58.4-65.4	56.8-64.2	
Prescriptions	Non hospitalization	55	82	66	124
	Hospitalization	85	60	70	205
	Overall	74	68	69	
	C.I.	70.8-77.1	65.0-72.0	65.4-72.2	
ICD	Non hospitalization	66	78	71	124
	Hospitalization	85	75	80	205
	Overall	78	76	77	
	C.I.	74.8-80.7	73.4-79.5	73.8-79.8	
CPT	Non hospitalization	66	76	71	124
	Hospitalization	84	77	80	205
	Overall	77	76	77	
	C.I.	74.4-80.3	73.3-79.5	73.7-79.7	
Lab	Non hospitalization	65	69	67	124
	Hospitalization	81	78	79	205
	Overall	75	74	75	
	C.I.	71.7-78.0	71.3-77.6	71.4-77.8	
Late fusion	Non hospitalization	80	66	73	124
	Hospitalization	81	90	86	205
	Overall	81	81	81	
	C.I.	78.4-84.0	78.4-84.0	77.8-83.5	
Early fusion	Non hospitalization	78	73	75	124
	Hospitalization	84	87	86	205
	Overall	**82**	**82**	**82**	
	C.I.	78.9-84.9	79.0-84.8	78.8-84.7	
Middle fusion	Non hospitalization	81	65	72	124
	Hospitalization	81	91	86	205
	Overall	81	81	81	
	C.I.	78.1-84.3	78.2-84.1	77.5-83.8	

Supplementary Table 8: Performance of the model on the white/ Caucasian population

		Precision	Recall	F-score	Support
Demographics	Non hospitalization	62	72	67	47
	Hospitalization	80	71	75	72
	Overall	73	71	72	
	C.I.	67.1-77.9	65.9-76.1	66.2-76.4	
Presscriptions	Non hospitalization	51	81	62	47
	Hospitalization	80	49	60	72
	Overall	68	61	61	
	C.I.	62.0-73.9	55.2-66.7	54.8-66.7	
ICD	Non hospitalization	78	77	77	47
	Hospitalization	85	86	86	72
	Overall	82	82	82	
	C.I.	77.5-87.8	77.1-87.5	77.2-87.6	
CPT	Non hospitalization	73	77	75	47
	Hospitalization	84	82	83	72
	Overall	80	80	80	
	C.I.	75.2-85.0	75.0-84.6	75.1-84.6	
Lab	Non hospitalization	68	68	68	47
	Hospitalization	79	79	79	72
	Overall	75	75	75	
	C.I.	69.6-80.2	69.4-80.0	69.5-80.0	
Late fusion	Non hospitalization	85	74	80	47
	Hospitalization	85	92	88	72
	Overall	85	85	85	
	C.I.	80.8-90.0	80.6-89.7	80.2-89.6	
Early fusion	Non hospitalization	83	81	82	47
	Hospitalization	88	89	88	72
	Overall	**86**	**86**	**86**	
	C.I.	81.8-90.2	81.7-90.0	81.7-90.0	
Middle fusion	Non hospitalization	77	70	73	47
	Hospitalization	82	86	84	72
	Overall	80	80	80	
	C.I.	75.0-85.0	75.3-85.0	74.9-84.8	

Supplementary Table 9: Performance of the model on the HISPANIC population

		Precision	Recall	F-score	Support
Demographics	Non hospitalization	25	9	13	11
	Hospitalization	76	91	83	34
	Overall	63	71	66	
	C.I.	49.3-79.1	62.1-80.8	54.0-77.2	
Prescriptions	Non hospitalization	33	73	46	11
	Hospitalization	86	53	65	34
	Overall	73	58	61	
	C.I.	63.0-83.6	47.8-66.7	50.9-69.3	
ICD	Non hospitalization	42	91	57	11
	Hospitalization	95	59	73	34
	Overall	82	67	69	
	C.I.	76.4-88.9	57.1-75.9	59.4-77.1	
CPT	Non hospitalization	39	64	48	11
	Hospitalization	85	68	75	34
	Overall	74	67	69	
	C.I.	65.1-84.5	56.7-75.0	59.6-76.6	
Lab	Non hospitalization	37	64	47	11
	Hospitalization	85	65	73	34
	Overall	73	64	67	
	C.I.	64.2-83.6	55.2-73.1	58.0-74.7	
Late fusion	Non hospitalization	42	45	43	11
	Hospitalization	82	79	81	34
	Overall	72	71	72	
	C.I.	62.5-82.3	62.5-79.3	61.9-80.0	
Early fusion	Non hospitalization	50	45	48	11
	Hospitalization	83	85	84	34
	Overall	**75**	**76**	**75**	
	C.I.	65.7-84.5	66.7-83.3	66.2-83.0	
Middle fusion	Non hospitalization	38	73	50	11
	Hospitalization	88	62	72	34
	Overall	75	64	67	
	C.I.	67.4-86.4	54.5-73.9	57.9-75.5	

Feature importance for individual EMR modalities:

Supplementary Figures 3-7 show feature importance from classifiers using individual EMR modalities (e.g., demographics, medication, CPT, comorbidities, and laboratory results) as predictor, instead of fusing all modalities together. Feature names are shown on y-axis while the x-axis indicate numeric value of feature importance from the classifier. Gender is the most important feature among the demographics. Medications related to the treatment of thyroid related diseases have the highest importance by medications-based classifier. Comorbidities related to the lungs and urinary systems are most important for the classifier based on comorbidities. For the CPT-based classifier, the most important features are from previous emergency department visits. Hemoglobin related laboratory tests have the highest weights in the laboratory results-based classifier.

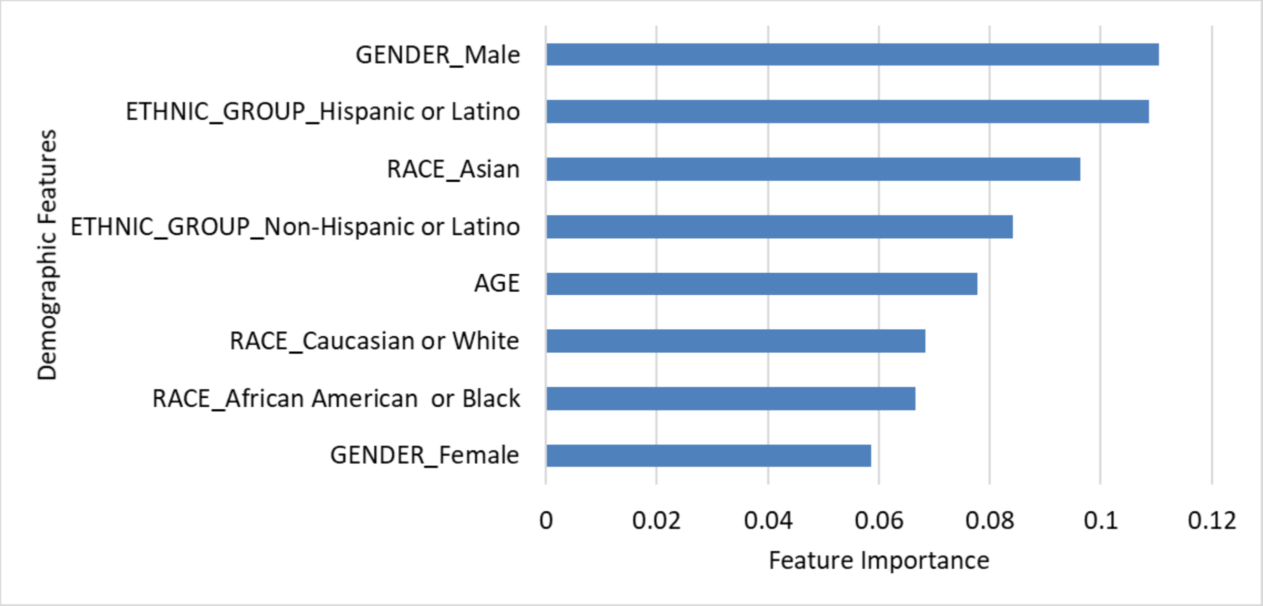

Supplementary Figure 3: Importance of demographic features for hospitalization prediction

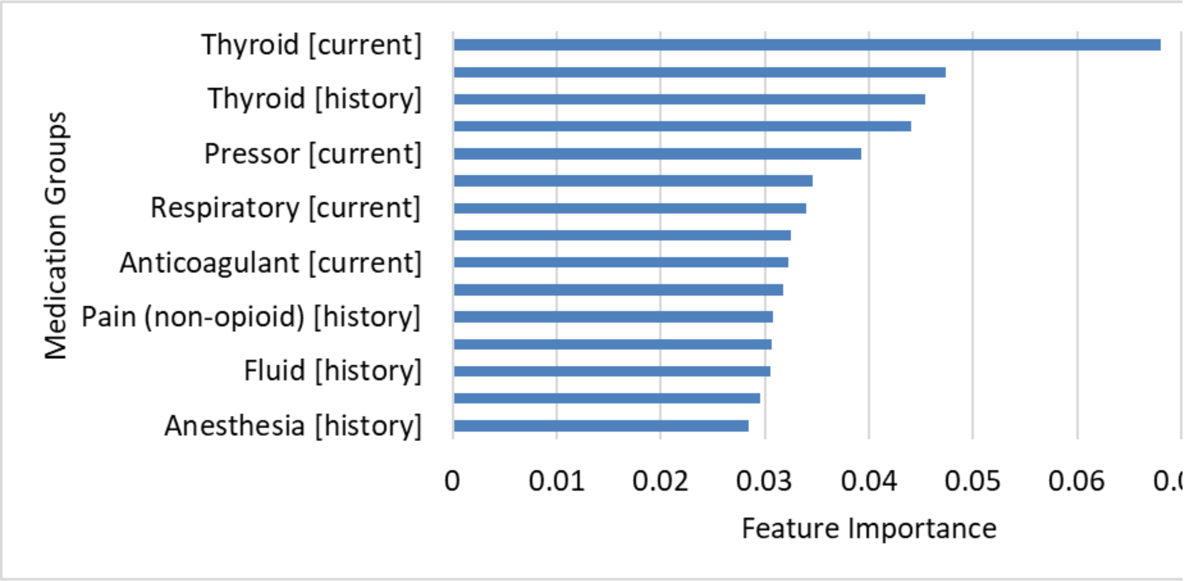

Supplementary Figure 4: Importance of medication groups for hospitalization prediction

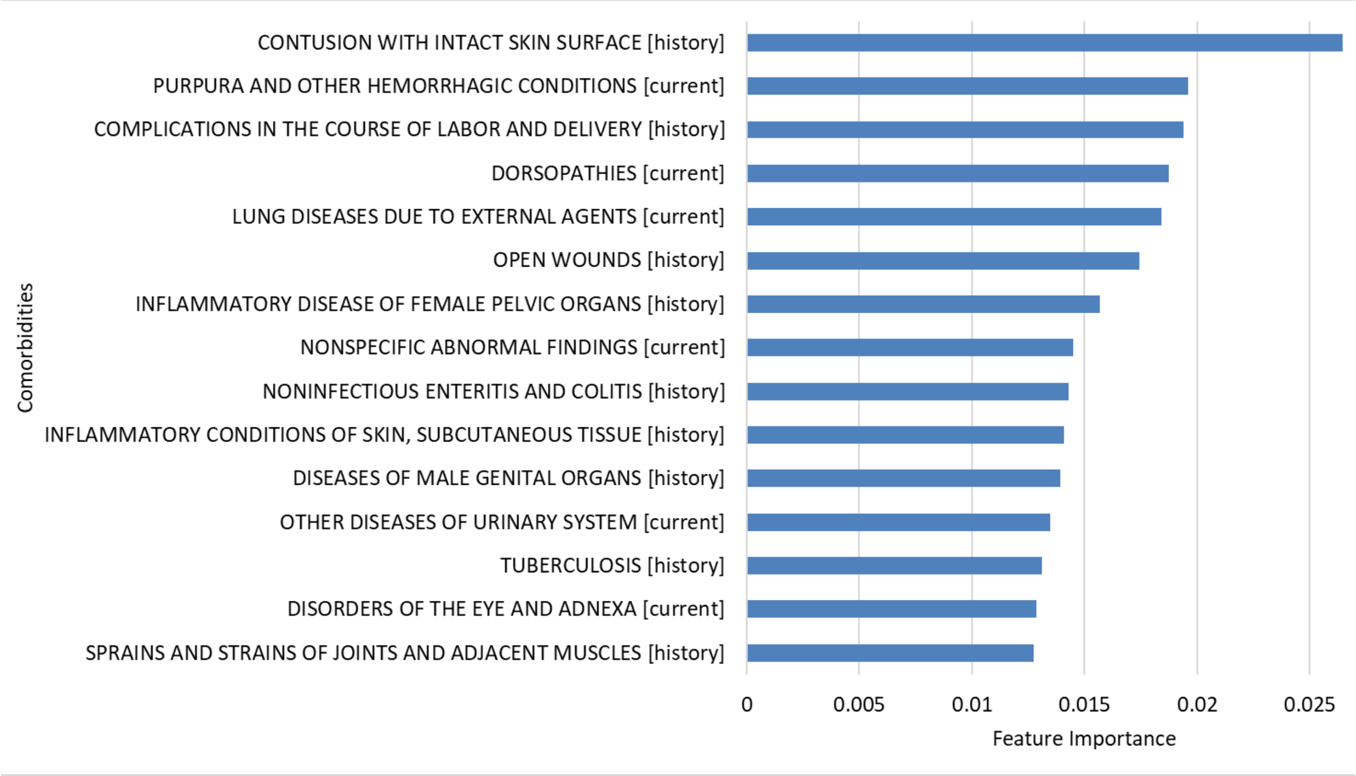

Supplementary Figure 5: Importance of comorbidities' groups for hospitalization prediction

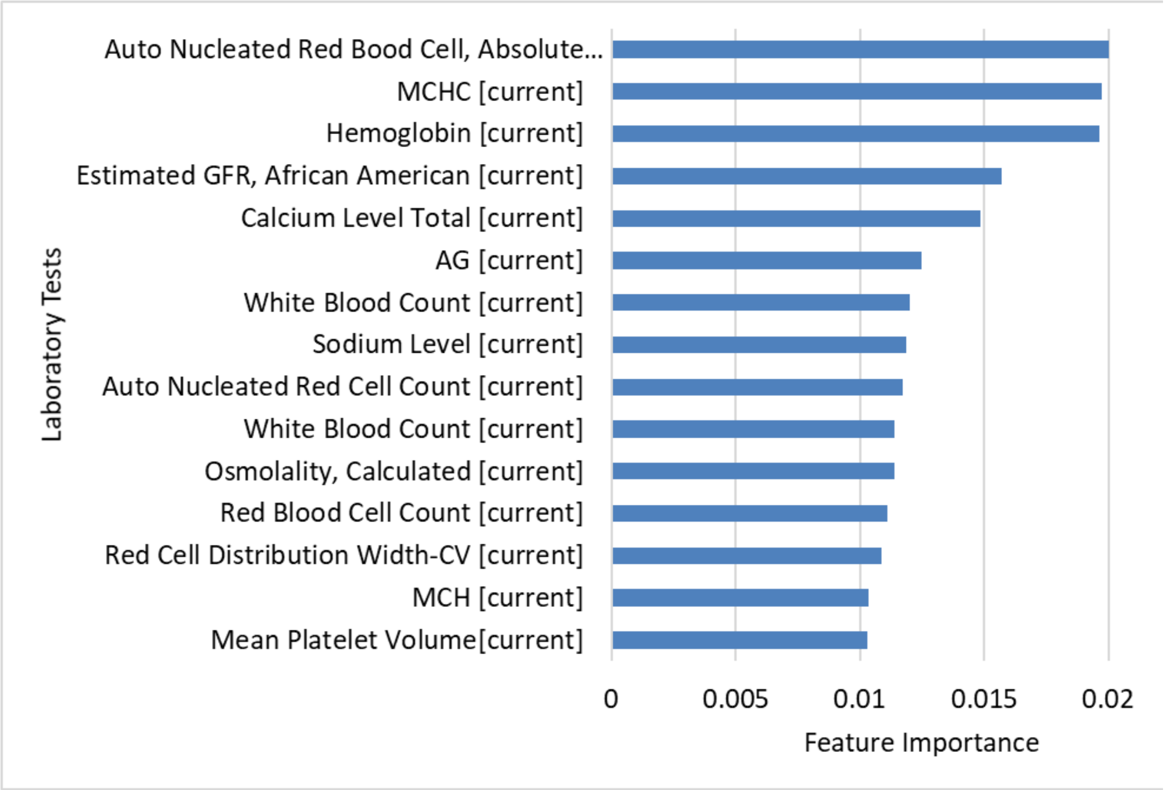

Supplementary Figure 6: Importance of laboratory test for hospitalization prediction

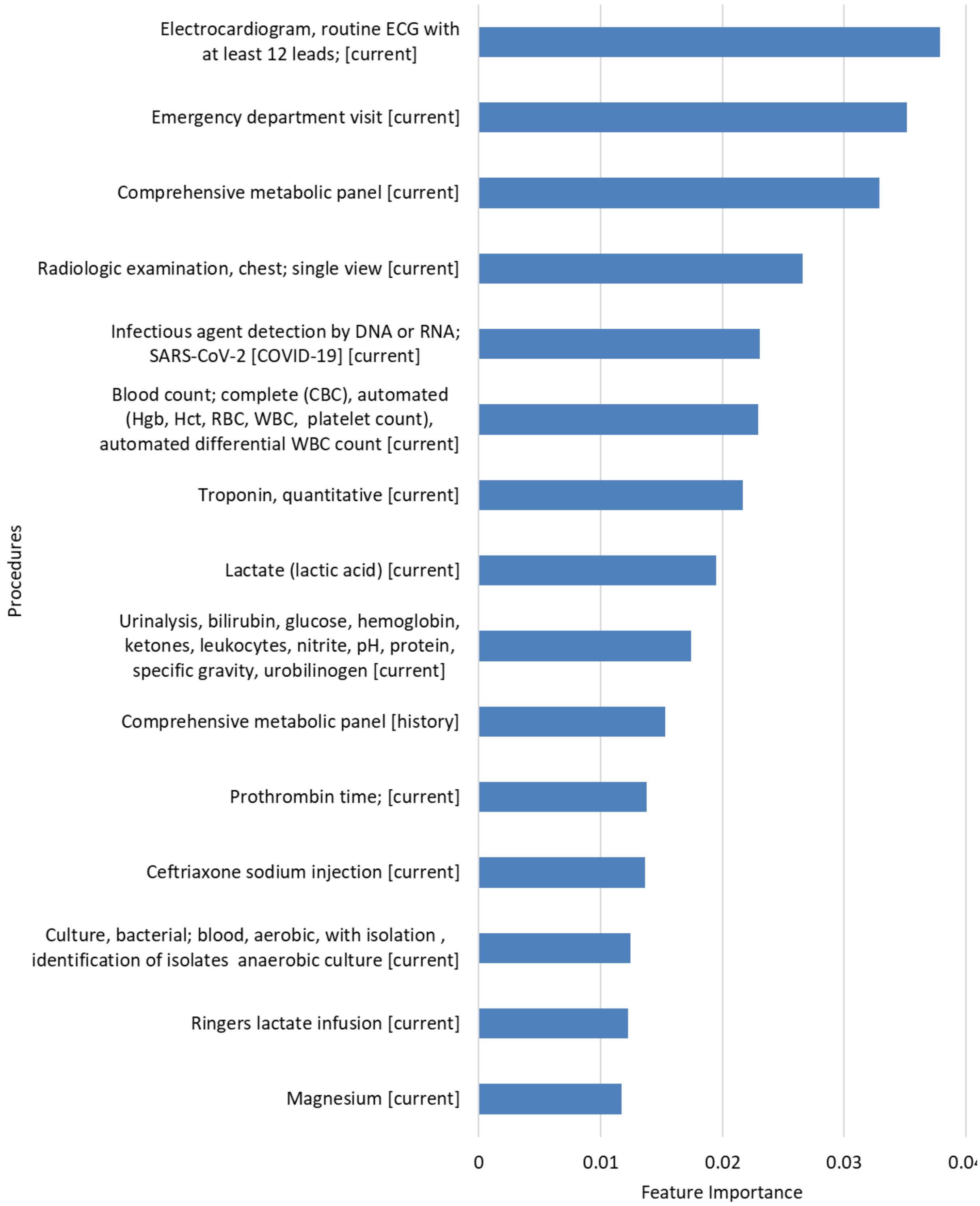

Supplementary Figure 7: Importance of CPT for hospitalization prediction

Supplementary References:

1. Wang, Z., et al., *Analysis of hospitalized COVID-19 patients in the Mount Sinai Health System using electronic medical records (EMR) reveals important prognostic factors for improved clinical outcomes.* medRxiv, 2020.
2. Obeid, J.S., et al., *An artificial intelligence approach to COVID-19 infection risk assessment in virtual visits: A case report.* Journal of the American Medical Informatics Association, 2020. **27**(8): p. 1321-1325.
3. Wagner, T., et al., *Augmented curation of clinical notes from a massive EHR system reveals symptoms of impending COVID-19 diagnosis.* Elife, 2020. **9**: p. e58227.
4. Shashikumar, S.P., et al., *Development and Prospective Validation of a Transparent Deep Learning Algorithm for Predicting Need for Mechanical Ventilation.* medRxiv, 2020.
5. Cheng, F.-Y., et al., *Using Machine Learning to Predict ICU Transfer in Hospitalized COVID-19 Patients.* Journal of Clinical Medicine, 2020. **9**(6): p. 1668.
6. Yu, C., et al., *Clinical Characteristics, Associated Factors, and Predicting COVID-19 Mortality Risk: A Retrospective Study in Wuhan, China.* American Journal of Preventive Medicine, 2020.
7. Levy, T.J., et al., *Development and Validation of a Survival Calculator for Hospitalized Patients with COVID-19.* medRxiv, 2020.
8. Vaid, A., et al., *Machine Learning to Predict Mortality and Critical Events in COVID-19 Positive New York City Patients: A Cohort Study.* Journal of Medical Internet Research, 2020.
9. Parchure, P., et al., *Development and validation of a machine learning-based prediction model for near-term in-hospital mortality among patients with COVID-19.* BMJ Supportive & Palliative Care, 2020.
10. Vaid, A., et al., *Federated Learning of Electronic Health Records Improves Mortality Prediction in Patients Hospitalized with COVID-19.* medRxiv, 2020.
11. Sun, H., et al., *CoVA: An Acuity Score for Outpatient Screening that Predicts COVID-19 Prognosis.* The Journal of infectious diseases, 2020.
12. Schalekamp, S., et al., *Model-based prediction of critical illness in hospitalized patients with COVID-19.* Radiology, 2020: p. 202723.
13. Banerjee, I., et al., *Development and performance of the pulmonary embolism result forecast model (PERFORM) for computed tomography clinical decision support.* JAMA network open, 2019. **2**(8): p. e198719-e198719.
